# Consumption of antibiotics in the community, European Union/European Economic Area, 1997–2017: data collection, management and analysis

**DOI:** 10.1093/jac/dkab171

**Published:** 2021-08-01

**Authors:** Robin Bruyndonckx, Niels Adriaenssens, Ann Versporten, Niel Hens, Dominique L Monnet, Geert Molenberghs, Herman Goossens, Klaus Weist, Samuel Coenen, the ESAC-Net study group, Reinhild Strauss, Eline Vandael, Stefana Sabtcheva, Marina Payerl-Pal, Isavella Kyriakidou, Jiří Vlček, Ute Wolff Sönksen, Elviira Linask, Emmi Sarvikivi, Philippe Cavalié, Marcel Feig, Flora Kontopidou, Ria Benkő, Gudrun Aspelund, Ajay Oza, Filomena Fortinguerra, Ieva Rutkovska, Jolanta Kuklytė, Marcel Bruch, Peter Zarb, Stephanie Natsch, Hege Salvesen Blix, Anna Olczak-Pieńkowska, Ana Silva, Ionel Iosif, Tomáš Tesař, Milan Čižman, Mayte Alonso Herreras, Vendela Bergfeldt, Susan Hopkins

**Affiliations:** 1 Laboratory of Medical Microbiology, Vaccine & Infectious Disease Institute (VAXINFECTIO), University of Antwerp, Antwerp, Belgium; 2 Interuniversity Institute for Biostatistics and statistical Bioinformatics (I-BIOSTAT), Data Science Institute, Hasselt University, Hasselt, Belgium; 3 Centre for General Practice, Department of Family Medicine & Population Health (FAMPOP), University of Antwerp, Antwerp, Belgium; 4 Centre for Health Economic Research and Modelling Infectious Diseases, Vaccine & Infectious Disease Institute (VAXINFECTIO), University of Antwerp, Antwerp, Belgium; 5 Disease Programmes Unit, European Centre for Disease Prevention and Control, Stockholm, Sweden; 6 Interuniversity Institute for Biostatistics and statistical Bioinformatics (I-BIOSTAT), Catholic University of Leuven, Leuven, Belgium

## Abstract

**Objectives:**

This article introduces a series of articles on antibiotic consumption in the community between 1997 and 2017, which provide an update of previous articles covering the periods 1997–2003 and 1997–2009.

**Methods:**

In this article, differences in participating countries, the ATC/DDD classification system, and data collection, validation and analysis between the current and previous series are described.

**Results:**

In the previous series, 33 European countries provided valid data for further analysis, while the current series focused on 30 countries belonging to the EU or the European Economic Area (EEA). For both series, data were collected in accordance with the WHO ATC classification system. While the previous series reported data in accordance with the ATC/DDD index 2011, the current series employed the ATC/DDD index 2019. Both series focused on consumption of antibacterials for systemic use (ATC J01) and collected data expressed in DDD per 1000 inhabitants per day and packages per 1000 inhabitants per day. When studying consumption expressed in packages per 1000 inhabitants per day, countries reporting total care data, i.e. community and hospital sector combined, were included in the previous series but excluded in the current series. While the previous series used non-linear mixed models to evaluate time trends in antibiotic consumption, the current series allowed for inclusion of change-points with a data-driven location. In addition, both series assessed the composition and quality of antibiotic consumption in the EU/EEA.

**Conclusions:**

The updated analyses of two decades of ESAC-Net data provide the most comprehensive and detailed description of antibiotic consumption in the community in Europe.

## Introduction

Antibiotics are medicines that are used to treat bacterial infections. Over time, their use and misuse have led to antimicrobial resistance resulting in treatment failure, increased costs of care and an elevated risk of mortality.^1^ In order to fight this worldwide public health problem, trustworthy information on antibiotic consumption is essential. In 2001, an international network of surveillance systems collecting comparable and reliable data on antimicrobial consumption in Europe — the European Surveillance of Antimicrobial Consumption (ESAC) project — was launched to accompany analogous surveillance on antimicrobial resistance.^2^ By 2009, the ESAC project had expanded to a network of 35 European countries, including 28 EU Member States, 3 European Economic Area (EEA)/European Free Trade Association countries (Iceland, Norway and Switzerland), a candidate country (North Macedonia) and 3 other countries (Israel, the Russian Federation and Turkey). In 2011, ECDC took over the coordination of the collection of data on antimicrobial consumption from EU/EEA countries as the European Surveillance of Antimicrobial Consumption Network (ESAC-Net),^3^ which covers all EU/EEA countries in agreement with Decision No. 1082/2013/EU of the European Parliament and the Council on serious cross-border threats to health.^4^ Information on antimicrobial consumption in the EU/EEA is publicly accessible through an interactive database and annual reports.^5^

This article introduces a series of articles on antibiotic consumption in the community in the EU/EEA, which provides an update of previous articles covering the periods 1997–2003^6–10^ and 1997–2009.^11–17^ In this series, we update the results of previously published brief reports focusing on antibacterials for systemic use, penicillins, cephalosporins, macrolides-lincosamides-streptogramins, quinolones, tetracyclines, sulphonamides and trimethoprim, and other antibacterials, as well as on indicators to assess the quality of antibiotic consumption in the community.^18–24^ We refer to community rather than outpatient or ambulatory consumption to stress our focus on primary care, and not hospital care, provided through prescriptions by general practitioners and other specialized doctors in community practices. Prescriptions in long-term care facilities and nursing homes, for outpatient parenteral antimicrobial therapy and in rehabilitation centres are all part of the community sector.^25^ We also add recent data and update the results applying the ATC/DDD index 2019,^26^ including relevant changes for the most commonly prescribed antibiotic substances. In addition, we have improved the longitudinal data analyses by allowing for change-points with a data-driven location.^27^

## Current (1997–2017) versus previous (1997–2009) series

Because several aspects regarding the data collection and analysis have changed when compared with the previous series of articles, we summarize the differences between the current and the previous series in the following paragraphs.

### Participants

**Figure 1.**
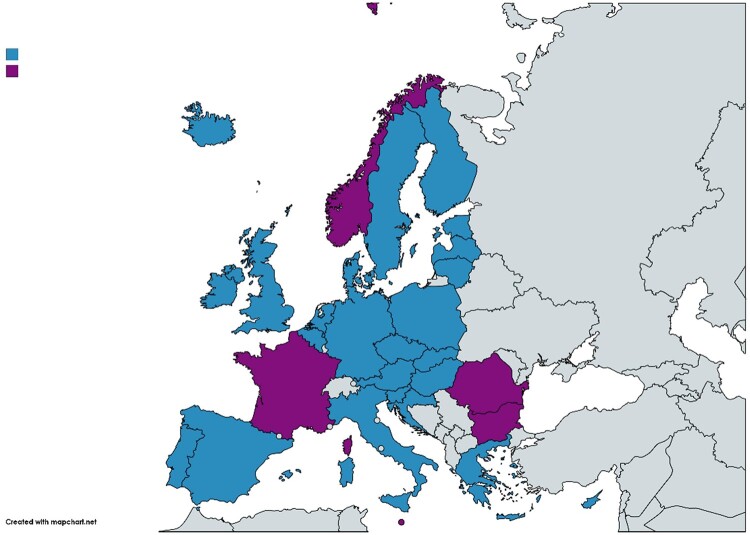
Map of Europe showing the 30 EU/EEA countries included in the series of articles. Blue colour indicates countries included in both the longitudinal (quarterly consumption) and the compositional (yearly consumption) data analysis, and purple colour indicates countries included only in the compositional data analysis. Map created using https://mapchart.net/europe.html.

In the previous series (1997–2009^11–17^), 33 European countries provided valid data for further analysis and all were included except North Macedonia and Turkey. In this series, we focused on the 30 countries belonging to the EU (28 countries) or the EEA (Iceland and Norway) that contributed data to ESAC-Net during 1997–2017 (Figure 1).

### ATC/DDD classification system

For both series of articles, data on the consumption of antibacterials for systemic use (ATC J01) in the community were collected in accordance with the WHO ATC classification. However, while the previous series reported data in accordance with the ATC/DDD index 2011,^28^ the current series reports data in accordance with the ATC/DDD index 2019.^26^ While the WHO aims to keep changes in DDDs to an absolute minimum, several important changes were implemented to better approximate the daily doses prescribed in practice. In 2017, the DDD changed for parenteral ampicillin/sulbactam (J01CR01, from 2 g to 6 g). In 2019, the DDD changed for parenteral ampicillin (J01CA01, from 2 g to 6 g), oral amoxicillin (J01CA04, from 1 g to 1.5 g), parenteral amoxicillin (J01CA04, from 1 g to 3 g), parenteral temocillin (J01CA17, from 2 g to 4 g), oral amoxicillin/clavulanic acid (J01CR02, from 1 g to 1.5 g), parenteral cefepime (J01DE01, from 2 g to 4 g), parenteral meropenem (J01DH02, from 2 g to 3 g), parenteral ciprofloxacin (J01MA02, from 0.5 g to 0.8 g), and parenteral colistin (J01XB01, from 3 MU to 9 MU). These changes (increases) in DDD values (e.g. for oral amoxicillin, from 1 g to 1.5 g) result in a lower number of DDDs per package (e.g. for oral amoxicillin, from 21 DDD to 14 DDD for a package containing 21 amoxicillin units of 1 g) and lower consumption when expressed in DDD per 1000 inhabitants per day.

Both series of articles focused on antibacterials for systemic use (ATC J01, excluding topical antibiotics), which consists of 10 groups (ATC-3 level) and 33 subgroups (ATC-4 level). While 229 unique chemical substances (ATC-5 level) were assigned an ATC code at the time the data for the previous series were collected, 259 unique chemical substances were assigned an ATC code at the time the data for the current series were collected.

### Data collection

The previous series of articles was based on data for 1997–2009 collected by the ESAC project, for which countries were asked to deliver data at product level, i.e. using a unique identifier for each of the medicinal product packages available in their country. Information on the number of packages consumed for each product had to be accompanied by an exhaustive and valid national register file including information on the number of DDD in each unique package.

The current series is based on data for 1997–2017 retrieved from The European Surveillance System (TESSy^29^) in which ESAC-Net data are stored (export date 26 June 2019). In this system, countries have two options for reporting antibiotic consumption data: either at product level or as aggregate numbers of DDDs consumed for each chemical substance (ATC-5 level) without the requirement to include information on package size. Data covering the period 1997–2009 were uploaded into TESSy after termination of the ESAC project.

When considering the most recent antibiotic consumption data, 2009 data were not reported for Switzerland in the previous series, and 2017 data were not reported for Czechia and Slovakia in the current series. In both series of articles, data from the last available year were used instead. In the previous series, 2004 data were used for Switzerland, and in the current series 2015 data were used for Czechia and 2016 data for Slovakia.

### Data description

Information on data source, coverage and, if available, online publication of national antibiotic consumption data were also collected (Table S1, available as Supplementary data at *JAC* Online). Most countries provided data from the same source in both series: 16 countries reported sales data, 6 reported reimbursement data and 2 reported data based on sales and reimbursement information (Italy and Slovenia). However, Croatia and Portugal reported sales data in the previous series and reimbursement data in the current series; Hungary, Poland and Romania reported reimbursement data in the previous series and sales data in the current series; and Spain reported reimbursement data in the previous series and data based on sales and reimbursement information in the current series. Sales data were based on reports from pharmaceutical companies, wholesalers, pharmacies or marketing research companies. Reimbursement data were collected by the third-party payer on the basis of financial claims from legitimate beneficiaries.

Antibiotic consumption was expressed in DDD per 1000 inhabitants per day, and if possible also in packages per 1000 inhabitants per day. Annual antibiotic consumption expressed in DDD per 1000 inhabitants per day was available for 30 countries overall and 28 countries in 2017 (all but Czechia and Slovakia). Annual antibiotic consumption expressed in packages per 1000 inhabitants per day was available for 25 countries overall (all but Germany, Hungary, Malta, Norway and Poland) and for 20 of these countries in 2017 (all but Czechia, Luxembourg, the Netherlands, Slovakia and the United Kingdom). The number of DDD per package was calculated by dividing antibiotic consumption expressed in DDD per 1000 inhabitants per day by antibiotic consumption expressed in packages per 1000 inhabitants per day for each subgroup (ATC-3 level). In this comparison, countries (or time-points) reporting total care data, i.e. aggregated consumption data reported for the community and hospital sector combined, were included in the previous series (e.g. Lithuania), but excluded in the current series (e.g. Romania, Bulgaria 1999–2005). Antibiotic consumption data are reported through annual data calls with the option to report quarterly data. Quarterly data expressed in DDD per 1000 inhabitants per day were available for 25 countries (all but Bulgaria, France, Malta, Norway and Romania).

In most of the participating countries, the denominator was provided as the WHO mid-year population in the previous series and by Eurostat in the current series.^30^ Some countries provided denominator data from their national statistical office (France, Germany, Portugal, Spain and Sweden for both series; Cyprus and the United Kingdom only for the previous series; Croatia, Czechia, Iceland, Lithuania and Slovenia only for the current series). Another possible source for denominator data was the insured population (Luxembourg for both series).

Data coverage for both series of articles was 100% for most countries. In the previous series, exceptions were Belgium (98%), Germany (90%), the Netherlands (90%), and Portugal (77%), and data for Luxembourg covered 100% of only the insured population. In the current series, exceptions were Belgium (99%), the Netherlands (93%) and Germany (85%), and data for Luxembourg covered 90% of only the insured population.

Some countries were only able to provide total care data, i.e. community and hospital sector combined. For all series including the current series, this concerned Bulgaria (up to 2005), Cyprus (between 2006 and 2017), Estonia (in 2001), Greece (between 2004 and 2010), Iceland (up to 2005 and between 2010 and 2013), Lithuania (up to 2011), Romania (between 2011 and 2017) and Slovakia (in 2011). These data were included because the vast majority of total care data represents community consumption (results not presented).

### Data validation

A software application was used to upload country data to the core database, i.e. ESAC Collect Manager Application for the previous series and The European Surveillance System (TESSy) for the current series. Both generated a standard validation report, which was sent for approval to country representatives, i.e. the ESAC lead national representative for the previous series and the ECDC National Focal Points for antimicrobial consumption and related Operational Contact Points nominated by the countries for the current series. More information on the data collection and validation can be found in the ESAC Yearbooks^31^ and in the ESAC-Net Reporting Protocols.^32^

### Analysis

As in the previous series,^11–16^ the EU/EEA ranking of consumption of antibiotics in the last reported year and expressed in DDD per 1000 inhabitants per day was presented. For countries that did not report antibiotic consumption in 2017, antibiotic consumption in the last available year was shown (2015 for Czechia and 2016 for Slovakia). If countries also reported antibiotic consumption expressed in packages per 1000 inhabitants per day, this ranking was presented as well. To facilitate a better understanding of the difference in these rankings, the number of DDD per package was presented. The evolution of the number of DDD per package over time was assessed using a linear mixed model, with the optimal model selected based on the Akaike Information Criterion.^33^^,^^34^

To evaluate the time trend and seasonal variation in antibiotic consumption from 1997 to 2017 in EU/EEA countries, quarterly data expressed in DDD per 1000 inhabitants per day were modelled with a non-linear mixed model including a sine wave, as applied and described in a tutorial article by Minalu *et al*.^35^ To improve model fit, we turned to a Bayesian framework that allows for the inclusion of change-points with a data-driven location in the current series.^36^ A practical overview of this approach, as well as its limitations, is provided in a tutorial article.^27^ Similar to the last series of articles, compositional data analysis was used to model annual antibiotic consumption expressed in DDD per 1000 inhabitants per day in order to assess trends of the relative proportions of the main antibiotic subgroups from 1997 up to 2017 for EU/EEA countries.^37^

In addition, the quality of antibiotic consumption in the community (EU/EEA countries, 2017) was assessed through 12 ESAC drug-specific quality indicators,^38^ as in the previous series.^17^ In the current series, an ECDC/EFSA/EMA joint scientific indicator was added.^39^

## Supplementary Material

dkab171_Supplementary_Data
