# Supplementary material for: Consumption of antibiotics in the community, European Union/European Economic Area, 1997–2017: data collection, management and analysis
Source: J Antimicrob Chemother. 2021 Aug 1;76(Suppl 2):ii2–6. doi: 10.1093/jac/dkab171 (PMC8314094; doi:10.1093/jac/dkab171)
Supplement: dkab171_Supplementary_Data [file dkab171_supplementary_data.docx]

**Supplementary data**

**Table S1 Overview of data type, coverage and data source per country for the last year antibiotic consumption data were reported, 2017 (except Slovakia – 2016 and Czechia – 2015).**

| Country | Data type | Coverage | Data source | DENOMINATOR SOURCE | NATIONAL DATA |
| --- | --- | --- | --- | --- | --- |
| Austria | Reimbursement | 100% | Health Insurance Company | Eurostat | https://www.ages.at/en/topics/ages-topics/antibiotics-resistances/ |
| Belgium | Reimbursement | 99% | Health Insurance Company | Eurostat | www.healthstat.be |
| Bulgaria | Sales | 100% | Marketing Research Company | Eurostat |  |
| Croatia | Reimbursement | 100% | Health Insurance Company | National Statistics Agency |  |
| Cyprus^‡^ | Sales | 100% | Ministry of Health | Eurostat |  |
| CzechIA | Reimbursement | 100% | Ministry of Health | National Statistics Agency |  |
| Denmark | Sales | 100% | Ministry of Health | Eurostat | https://www.danmap.org |
| Estonia | Sales | 100% | Medicines Agency | Eurostat |  |
| Finland | Sales | 100% | Medicines Agency | Eurostat |  |
| France | Sales | 100% | Medicines Agency | National Statistics Agency |  |
| Germany | Reimbursement | 85% | Health Insurance Company | National Statistics Agency |  |
| Greece | Sales | 100% | Medicines Agency | Eurostat |  |
| Hungary | Sales | 100% | Marketing Research Company | Eurostat |  |
| Iceland | Sales | 100% | Community pharmacists | National Statistics Agency |  |
| Ireland | Sales | 100% | Marketing Research Company | Eurostat |  |
| Italy | Both | 100% | Medicines Agency | Other source | <https://www.aifa.gov.it/l-uso-degli-antibiotici-in-italia%C2%A0> |
| Latvia | Sales | 100% | Medicines Agency | Eurostat | https://www.zva.gov.lv/en/news-and-publications/publications/statistics-medicines-consumption |
| Lithuania | Sales | 100% | Medicines Agency | National Statistics Agency |  |
| Luxembourg | Reimbursement | 90% | Health Insurance Company | Health Insurance Company |  |
| Malta | Sales | 100% | Ministry for Health | Eurostat |  |
| Netherlands | Sales | 93% | Community pharmacists | Other source |  |
| Norway | Sales | 100% | National Institute | Other source | <http://www.norpd.no/> |
| Poland | Sales | 100% | Marketing Research Company | Eurostat | www.antybiotyki.edu.pl |
| Portugal | Reimbursement | 100% | Medicines Agency | National Statistics Agency |  |
| Romania^‡^ | Sales | 100% | Marketing Research Company | Eurostat |  |
| Slovakia | Sales | 100% | Medicines Agency | Eurostat |  |
| Slovenia | Both | 100% | National Institute | National Statistics Agency | http://www.si-map.org |
| Spain | Both | 100% | Ministry of Health and Marketing Research Company | National Statistics Agency | http://www.resistenciaantibioticos.es/es/profesionales/vigilancia/mapas-de-consumo |
| Sweden | Sales | 100% | Community pharmacists | National Statistics Agency | <https://www.folkhalsomyndigheten.se/publicerat-material/publikationsarkiv/s/swedres-svarm-2019/> |
| United kingdom | Reimbursement | 100% | Community pharmacists | Eurostat |  |

^‡^: Total care. i.e. community and hospital sector combined
